# Fallbericht: Persistierendes Fieber nach SARS-CoV-2-Infektion

**DOI:** 10.1007/s00108-022-01315-x

**Published:** 2022-03-17

**Authors:** T. Kliem, D. Strobel, F. Heinke, M. Pavel, M. F. Neurath, C. Neufert

**Affiliations:** 1 Medizinische Klinik 1, Universitätsklinikum Erlangen, Ulmenweg 18, 91054 Erlangen, Deutschland; 2 Deutsches Zentrum Immuntherapie, Erlangen, Deutschland

**Keywords:** Subakute Thyreoiditis, Thyreoiditis de Quervain, COVID-19, Diagnostischer Ultraschall, Tachykardie, Subacute granulomatous thyroiditis, De Quervain’s thyroiditis, COVID-19, Diagnostic ultrasound, Tachycardia

## Abstract

Ein 44-jähriger Patient hatte anhaltendes Fieber und eine deutliche Erhöhung des C-reaktiven Proteins (CRP) nach einer Infektion mit dem *severe acute respiratory syndrome coronavirus type 2* (SARS-CoV-2) Bei zunehmender Sinustachykardie erfolgte die Bestimmung der Schilddrüsenhormone, welche eine Hyperthyreose zeigten. Sonographisch fand sich eine Vergrößerung der Schilddrüse mit unscharf begrenzten echoarmen Arealen. Wir stellten die Diagnose einer SARS-CoV-2-Infektions-assoziierten subakuten Thyreoiditis und leiteten eine Therapie mit Prednisolon ein. Diese Therapie führte kurzfristig zu einer Besserung des klinischen Zustands und zu einer vollständigen Ausheilung binnen drei Monaten.

## Abstract (en)

A 44-year-old man had persistent fever following a severe acute respiratory syndrome coronavirus type 2 (SARS-CoV-2) infection. Because of progressive sinus tachycardia, thyroid hormones were measured which showed hyperthyroidism. Thyroid sonography revealed enlargement of the thyroid gland with hypoechoic areas with blurred margins. We diagnosed subacute granulomatous thyroiditis associated with SARS-CoV‑2 infection and initiated therapy with prednisolone. This therapy resulted in rapid improvement of the patient’s clinical condition and complete remission after three months.

## Anamnese

Die stationäre Aufnahme eines 44-jährigen pflegebedürftigen Patienten erfolgte bei SARS-CoV-2-Infektion mit moderater Symptomatik (Schüttelfrost sowie körperliche Abgeschlagenheit). Der erste positive Abstrich auf SARS-CoV-2-RNA war acht Tage zuvor erfolgt. Als Vorerkrankungen bestanden ein frühkindlicher Hydrozephalus mit ventrikuloperitonealer Shuntversorgung bei Z. n. CMV-Enzephalitis, eine arterielle Hypertonie und eine Epilepsie.

## Befunde

Bei Aufnahme präsentierte sich ein wacher, orientierter und kardiorespiratorisch stabiler Patient ohne zusätzlichen Sauerstoffbedarf. In der körperlichen Untersuchung waren eine subfebrile Körpertemperatur (38,3 °C), eine Tachykardie (105/min) und eine Amaurosis (bei Z. n. CMV-Infektion) auffällig. Im Labor imponierten eine Leukopenie (3060/µl, Ref. 4000–10.000/µl) sowie ein erhöhtes CRP (100,4 mg/l, Ref. <5 mg/l). Im Rachenabstrich ließ sich die SARS-CoV-2-Variante B.1.1.7 nachweisen (Ct-Wert 24,8).

Im stationären Verlauf traten für knapp drei Wochen regelmäßig Fieberschübe bis maximal 39,4 °C auf. Dabei undulierte das CRP auf einem deutlich erhöhten Niveau um ca. 120 mg/l und stieg schließlich bis auf 193,9 mg/l an. Der Patient berichtete hierunter lediglich über eine ausgeprägte Erschöpfung. Diese Befunde wurden zunächst im Kontext der SARS-CoV-2-Infektion bzw. einer möglichen Superinfektion interpretiert, wobei das Procalcitonin im Referenzbereich lag und mehrfache Blutkulturen negativ blieben. Weitere diagnostische Maßnahmen erbrachten zunächst keine klärenden Befunde. So zeigte ein Thorax-CT nur geringe COVID-typische bipulmonale Milchglasinfiltrate (<10 % des Lungenparenchyms). In der Sonographie des Abdomens fanden sich eine Steingallenblase sowie Nierenzysten bds. ohne Zeichen einer akuten Infektion. In einer Liquorpunktion bot sich kein Anhalt für eine Shuntinfektion und CMV-DNA war (bei Z. n. schwerer CMV-Infektion) im Blut nicht detektierbar. Schließlich fand sich in einer Urinkultur *Klebsiella pneumoniae* in erhöhter Keimzahl (>100.000/ml), jedoch ohne relevante Leukozyturie (<10.000/ml). Bei fehlendem alternativem Infektfokus erfolgte eine antibiogrammgerechte Therapie mit Piperacillin/Tazobactam, welche jedoch nicht zu einer Beendigung der Fieberschübe oder einem Abfall des CRP führte.

**Figure 1:**
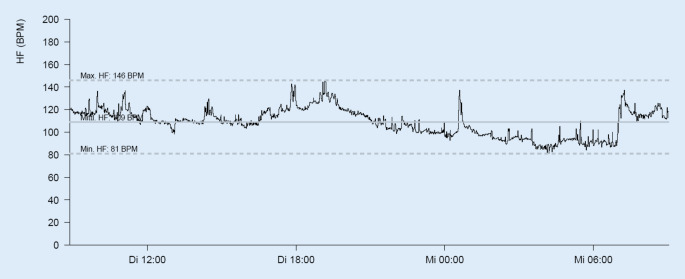

Im Verlauf fiel eine progrediente Ruhetachykardie auf, die sich im 24 h-EKG als Sinustachykardie darstellte (Herzfrequenz: im Mittel 109/min, maximal 146/min; Abb. 1). Daraufhin erfolgte die Bestimmung der Schilddrüsenwerte, die ein supprimiertes TSH (0,01 mIU/l) sowie deutlich erhöhte Schilddrüsenhormone (fT_3_ 11,77 pmol/l, fT_4_ 27,48 pmol/l) ergab (Tab. 1).

**Table 1:** 

Parameter	Diagnosestellung (05.05.2021)	5 Tage nach Therapiebeginn Prednisolon (11.05.2021)	4 Wochen nach Beendigung Prednisolontherapie (19.08.2021)	Referenzbereiche
TSH (mIU/l)	0,01	0,03	3,41	0,2–4,0
fT_3_ (pmol/l)	11,77	7,30	5,07	3,2–7,2
fT_4_ (pmol/l)	27,48	28,72	18,5	11,5–24,0
CRP (mg/l)	193,9	26,2	4,2	<5,0

*TSH* Thyreoidea-stimulierendes Hormon, *fT3* freies Trijodthyronin, *fT4* freies Thyroxin, *CRP* C-reaktives Protein

**Figure 2:**
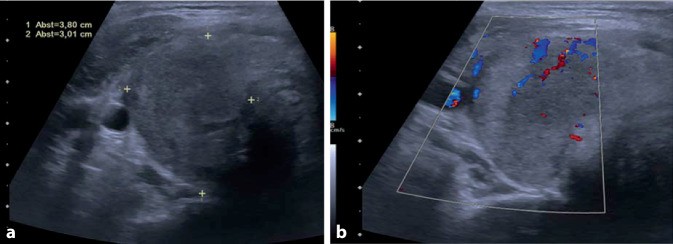

Zur Differenzialdiagnose der Hyperthyreose erfolgte eine Sonographie des Halses. Diese zeigte eine rechtsbetonte Vergrößerung der Schilddrüse (Gesamtvolumen 45,2 ml) mit unscharf begrenzten echoarmen Arealen ohne Hypervaskularisation (Abb. 2). Insgesamt war das sonographische Bild gut vereinbar mit einer subakuten Thyreoiditis. Außerdem erfolgte die serologische Bestimmung von Antikörpern, die unauffällige Werte für Anti-TPO, Anti-TSH-Rezeptor-Antikörper und Anti-Thyreoglobulin erbrachte. In Zusammenschau der Befunde stellten wir die Diagnose einer postinfektiösen subakuten Thyreoiditis, getriggert durch eine SARS-CoV-2-Infektion.

## Diagnose

- SARS-CoV-2-Infektions-assoziierte subakute Thyreoiditis (Thyreoiditis de Quervain)

## Therapie und Verlauf

**Figure 3:**
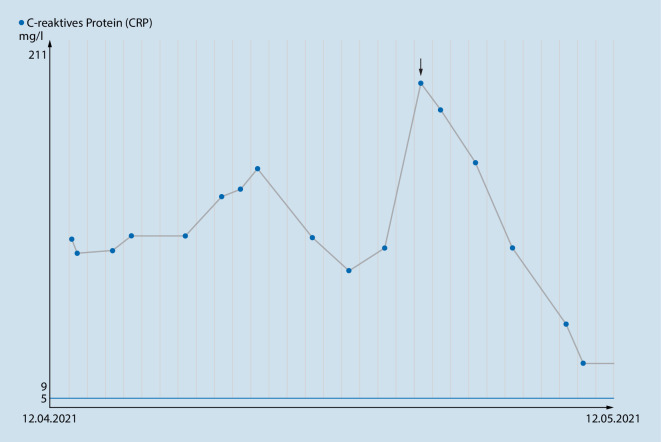

Nach Diagnosestellung wurde eine Therapie mit Prednisolon eingeleitet (initial 50 mg täglich für sieben Tage, anschließend stufenweises Ausschleichen über 10 Wochen). Nach Therapiebeginn besserte sich der klinische Zustand des Patienten rasch und nachhaltig. Es traten keine weiteren Fieberschübe auf und die Herzfrequenz normalisierte sich. Das CRP war deutlich rückläufig und lag bei stationärer Entlassung – fünf Tage nach Therapiebeginn – bei 26,2 mg/l (Abb. 3; Tab. 1).

**Figure 4:**
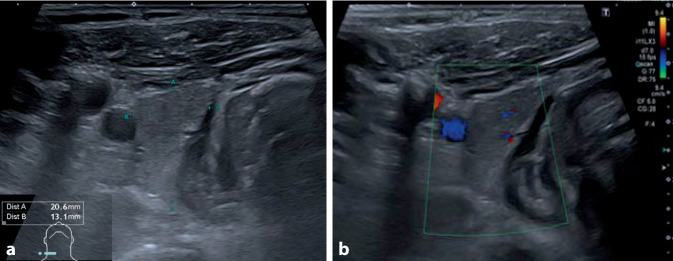

In einer ambulanten Kontrolle in unserer Klinik nach etwa drei Monaten zeigte sich der Patient beschwerdefrei. Die Therapie mit Prednisolon war wie geplant vier Wochen zuvor beendet worden. Die Sonographie der Schilddrüse ergab eine normale Organgröße (Gesamtvolumen 11 ml) und ein homogenes echonormales Binnenreflexmuster ohne Nachweis von fokalen Läsionen (Abb. 4). Im Labor zeigten sich eine euthyreote Stoffwechsellage sowie ein normwertiges CRP (Tab. 1). Somit lag eine vollständige Ausheilung der subakuten Thyreoiditis vor.

## Diskussion

Die subakute Thyreoiditis betrifft meist Erwachsene mittleren Alters im Anschluss an eine Infektion der oberen Atemwege (Symptombeginn nach 2–8 Wochen). Pathophysiologisch wird eine postvirale, zytokinvermittelte Entzündungsreaktion angenommen, welche zur Zerstörung von Schilddrüsenfollikeln und zur unkontrollierten Freisetzung von Schilddrüsenhormonen führt [1]. Häufigstes, aber nicht obligates Symptom sind Schmerzen im Halsbereich mit druckempfindlicher Schilddrüse (etwa 95 % der Fälle; [2]). Typisch sind weiterhin Fieber, Abgeschlagenheit, hyperthyreoseassoziierte Befunde (Palpitationen, Gewichtsverlust, vermehrtes Schwitzen) sowie einer Hyperthyreose entsprechende Laborbefunde und ein erhöhtes CRP [3]. Sonographisch findet sich charakteristischerweise eine vergrößerte Schilddrüse mit landkartenartigen hypoechogenen Arealen. Im Gegensatz zur Hyperthyreose bei Morbus Basedow lässt sich farbdopplersonographisch keine Hypervaskularisation nachweisen [4, 5]. Zur Therapie wird neben nichtsteroidalen Analgetika, v. a. bei systemischen Symptomen, eine antiinflammatorische Therapie mit Prednisolon empfohlen [2]. Meist kommt es im Verlauf zur Ausheilung.

Nach Infektion mit SARS-CoV‑2 wurde von mehreren Fällen einer subakuten Thyreoiditis berichtet [6]. Das mediane Alter der Betroffenen betrug 40 Jahre und über 70 % waren Frauen, wobei der Abstand zwischen Beginn der respiratorischen Symptome und der Diagnose einer subakuten Thyreoiditis bei 5–49 Tagen (Median 26 Tage) lag. Schmerzen in der Halsregion wurden mit 81 % etwas seltener berichtet als bei nicht mit SARS-CoV‑2 assoziierten Fällen. Stets ließen sich jedoch die oben beschriebenen typischen Ultraschallbefunde nachweisen [6].

Fieber ist ein Leitsymptom einer SARS-CoV-2-Infektion. Bei prolongiertem Fieber, insbesondere ohne begleitende respiratorische Symptomatik und ohne Hinweis auf eine Sekundärinfektion, sollten infektiöse und nichtinfektiöse Differenzialdiagnosen in Betracht gezogen werden. Im dargestellten Fall kamen bei einliegendem Fremdmaterial, Z. n. nach CMV-Infektion sowie Nachweis pathogener Erreger im Urin mehrere infektiöse Fieberursachen unmittelbar in Betracht. Dagegen lag der für eine subakute Thyreoiditis typische Befund einer druckschmerzhaften Schilddrüse – so wie in ca. 20 % der bisher berichteten SARS-CoV-2-assoziierten Fälle – bei unserem Patienten nicht vor. Jedoch lenkte die Sinustachykardie den Verdacht auf eine Hyperthyreose und war gemeinsam mit der Sonographie Schlüssel zur Diagnose.

Bei persistierendem Fieber bzw. CRP-Erhöhung nach SARS-CoV-2-Infektion sollte, auch bei fehlendem typischem Lokalbefund, eine subakute Thyreoiditis als Ursache in Betracht gezogen werden.

## Fazit für die Praxis

- Die subakute Thyreoiditis kann auch in Folge einer SARS-CoV-2-Infektion auftreten und sollte als Differenzialdiagnose entsprechend berücksichtigt werden.
- Häufigste Symptome sind Schmerzen in der Halsregion, Fieber, Abgeschlagenheit, Tachykardie sowie weitere Zeichen einer hyperthyreoten Stoffwechsellage.
- Die Schilddrüsensonographie mit typischem Befund kann, insbesondere beim Fehlen einer druckschmerzhaften Halsregion, die Diagnosestellung ermöglichen.
